# A Thermostable Monoacylglycerol Lipase from Marine *Geobacillus* sp. 12AMOR1: Biochemical Characterization and Mutagenesis Study

**DOI:** 10.3390/ijms20030780

**Published:** 2019-02-12

**Authors:** Wei Tang, Dongming Lan, Zexin Zhao, Shuang Li, Xiuting Li, Yonghua Wang

**Affiliations:** 1 School of Food Science and Engineering, Guangdong Research Center of Lipid Science and Applied Engineering Technology, State Key Laboratory of Pulp and Paper Engineering, South China University of Technology, Guangzhou 510641, China; 201620119718@mail.scut.edu.cn (W.T.); dmlan@scut.edu.cn (D.L.); lishuang6991@163.com (S.L.); 2 School of Biology and Biological Engineering, South China University of Technology, Guangzhou 510006, China; bizexin-zhao@mail.scut.edu.cn; 3 Beijing Advanced Innovation Center for Food Nutrition and Human Health, Beijing Technology and Business University (BTBU), Beijing 100048, China; lixt@btbu.edu.cn

**Keywords:** monoacylglycerol lipase, marine *Geobacillus* sp., thermostability, mutagenesis study, substrate selectivity

## Abstract

Lipases with unique substrate specificity are highly desired in biotechnological applications. In this study, a putative marine *Geobacillus* sp. monoacylglycerol lipase (GMGL) encoded gene was identified by a genomic mining strategy. The gene was expressed in *Escherichia coli* as a His-tag fusion protein and purified by affinity chromatography with a yield of 264 mg per liter fermentation broth. The recombinant GMGL shows the highest hydrolysis activity at 60 °C and pH 8.0, and the half-life was 60 min at 70 °C. The GMGL is active on monoacylglycerol (MAG) substrate but not diacylglycerol (DAG) or triacylglycerol (TAG), and produces MAG as the single product in the esterification reaction. Modeling structure analysis showed that the catalytic triad is formed by Ser97, Asp196 and His226, and the flexible cap region is constituted by residues from Ala120 to Thr160. A mutagenesis study on Leu142, Ile145 and Ile170 located in the substrate binding tunnel revealed that these residues were related with its substrate specificity. The k_cat_/K_m_ value toward the pNP-C6 substrate in mutants Leu142Ala, Ile145Ala and Ile170Phe increased to 2.3-, 1.4- and 2.2-fold as compared to that of the wild type, respectively.

## 1. Introduction

Lipases are versatile enzymes that can catalyze various kinds of reactions, such as hydrolysis, esterification, interesterification, and transesterification. They have been widely used in industry of food, energy, fine chemical and pharmaceutical. In particular, lipases with strict substrate specificity are highly desired since they can product high content of target compounds without any by-product. Until now, most lipases were reported as triacylglycerol (TAG) hydrolyzing lipase, which will produce a mixture product of TAG, diacylglycerol (DAG) and monoacylglycerol (MAG) in the esterification reaction using glycerol and fatty acid as substrates. Among them, DAG and MAG have been reported to have significant values in human dietary nutrition [1,2] and can be used as food emulsifiers. Enzymatic synthesis of DAG and MAG were more promising as compared to the chemical synthesis strategy in industrial applications [3]. However, it should need an additional separate procedure to obtain a single product of DAG or MAG from oil, which will consume more cost and time. 

Monoacylglycerol lipases (MGLs) are a subclass of lipolytic enzymes which are able to catalyze the hydrolysis of MAG but not TAG and DAG substrates [4,5]. Recently, crystal structures of MGLs from human, bacterial, yeast and *Mycobacterium tuberculosis* have been resolved [6,7,8]. All these structures possessed a common β-sheet core region surrounded by α helices and loops, despite low sequence identity. Besides the highly conserved region, MGLs contained a flexible structural feature, named cap domain [9,10,11]. Crystal structures not only provide more insights on understand the catalytic mechanism of MGLs but also pave a way to tailor the MGLs to fulfill the requirement for biotechnological applications.

Most current commercial lipases were isolated from environmental microorganisms which have the advantages of good tolerance against heat or other stresses, high activity in various reaction conditions, and wide substrate scope with high enantiomeric selectivity and/or stereoselectivity [12,13]. Marine ecosystems are a vast repository for discovering industrially useful biocatalysts [14], but microorganisms from such environments are not easy to culture at the laboratory condition, limiting the enzymes discovery. Fortunately, thousands of genomic data of marine microorganisms or metagenomic data are available [15,16,17]. Thus, genomic mining seems to be a feasible strategy for discovering valuable enzymes with industrial potential.

In this study, a putative monoglyceride lipase gene sequence was identified from the genome of marine *Geobacillus* sp. 12AMOR1 [18]. The gene was expressed in *Escherichia coli* and its recombinant protein was purified by affinity chromatography. Biochemical characterization, structural modeling and a mutagenesis study of GMGL were conducted, which provided basic knowledge on enzymes from marine sources.

## 2. Results and Discussion

### 2.1. Gene Sequence Identification and Recombinant Protein Production

**Figure 1:**
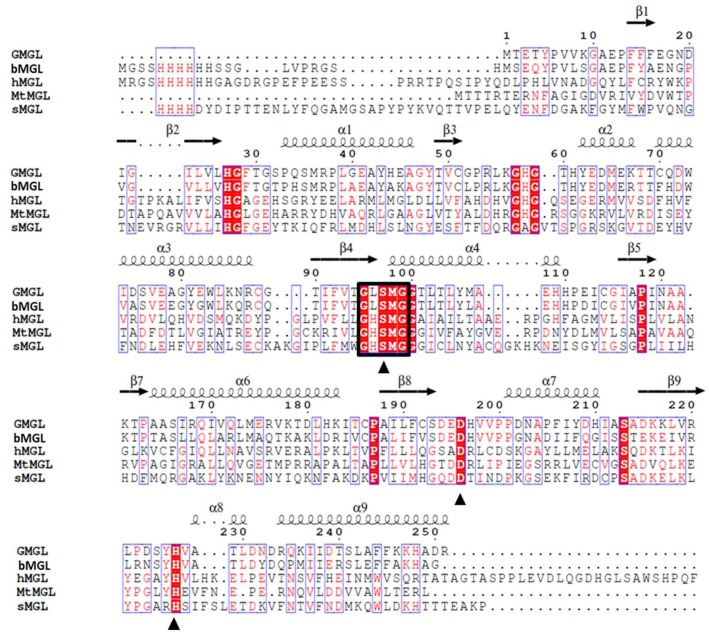
Sequence alignment of MGLs. The sequence derived from GMGL (monoacylglycerol lipase from *Geobacillus* sp., this study), *bMGL* (PDB: 3RM3, Bacterial), *hMGL* (PDB: 3HJU, Human), *sMGL* (PDB:4ZXF, yeast) and *MtMGL* (PDB:6EIC, *Mycobacterium tuberculosis*). The pentapeptide motif in all the sequence is marked with black frame. The putative catalytic triads were indicated with black triangle.

Thermophilic *Geobacillus* sp. 12AMOR1 was collected from an Arctic deep-sea hydrothermal vent site sample and its genome has been completely sequenced [18]. According to the conserved G-X-S-Q-G pentapeptide motif containing sequence searching, there are three putative lipolytic enzymes sequence found in the draft genome sequence of marine *Geobacillus* sp. Among them, a putative monoacylglycerol lipase gene interested us due to it shared 67% sequence identity with that of monoacylglycerol lipase from bacterium (bMGL), implying that it may have activity toward MAG substrate. But it showed very low sequence identity, 18%, 22% and 19%, to MGLs from human, yeast and *Mycobacterium tuberculosis*, respectively (Figure 1). 

**Figure 2:**
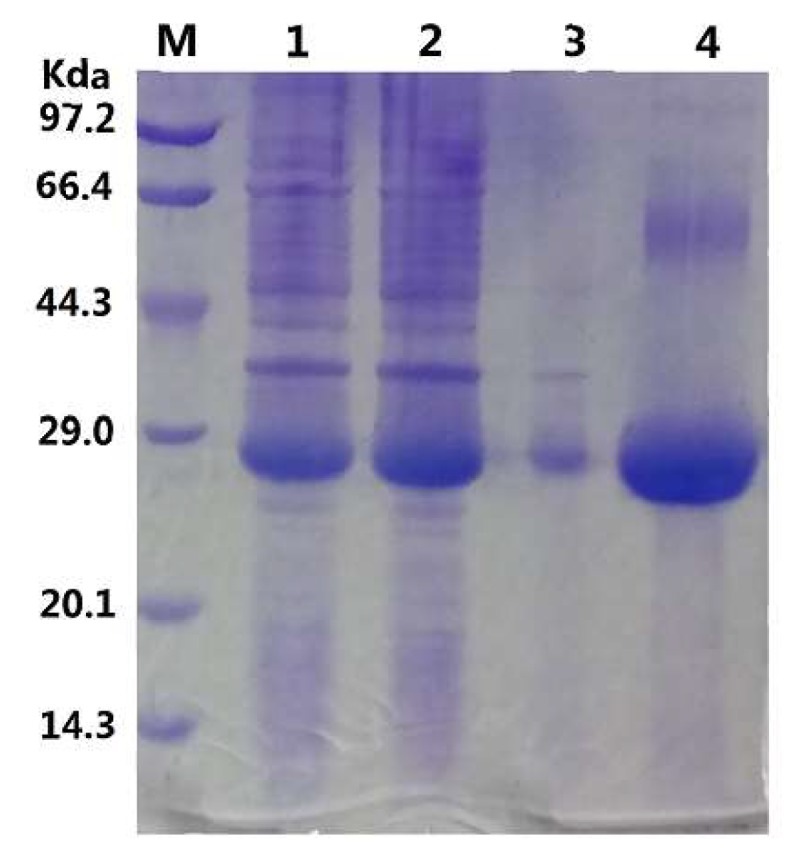
Sodium dodecyl sulfate polyacrylamide gel electrophoresis (SDS-PAGE) analysis of expression and purification of GMGL. Lane M: molecular weight marker. Lane 1: whole cell proteins; Lane 2: supernatant of cell lysate after centrifugation; Lane 3: pellet of cell lysate after centrifugation; Lane 4: purified GMGL.

To assess whether this putative monoacylglycerol lipase has the lipolytic activity, the gene was obtained by artificial synthesis strategy and expressed as a His-tag fusion protein in *E. coli* strain BL21 (DE3). As shown in Figure 2, recombinant GMGL was highly expressed and existed in the soluble fraction of cell lysates. Purified GMGL appears as a band at about 28 kDa which correlated well to its predicted molecular weight. The production and purification of GMGL are summarized in Table 1. An amount of 264 mg purified GMGL with a total activity of 163,812 U can be obtained from one liter fermentation broth by Ni+ affinity chromatography, which provided enough enzymes for our subsequent biochemical characterization and application evaluation.

### 2.2. Biochemical Characterization of Monoacylglycerol Lipase from *Geobacillus* sp. (GMGL)

#### 2.2.1. Effect of Temperature on Lipase Activity and Thermostability

**Figure 3:**
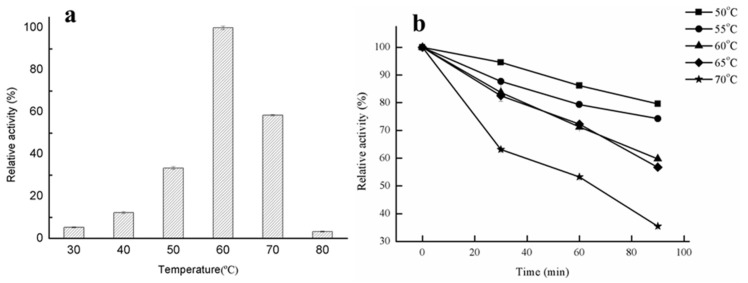
Effects of temperature on lipase activity and stability. (**a**) Effect of temperature on lipase activity. The enzyme activities were measured at various temperatures using glycerin monostearate as substrate. Activity obtained at 60 °C was taken as 100%. (**b**) Analysis of GMGL thermostability after incubating the enzyme under various temperatures (from 50 to 70 °C). The activities were measured at every 30 min interval, and the initial activity was taken as 100%.

The effects of temperature on lipase activity and the theromostability of GMGL at different temperatures were determined using glycerin monostearate as substrate. As shown in Figure 3a, GMGL showed the highest activity at 60 °C, while it still maintained above 50% activity at 70 °C. The thermostability of recombinant GMGL was investigated by incubation at relatively high temperature (50–70 °C). As shown in Figure 3b, GMGL has high tolerance against heat treatment. It retained more than 70% of the original activity after an incubation at 55 °C for 90 min, and the half-life of GMGL at 70 °C was 60 min, indicating that GMGL was a thermostable enzyme with potential in industrial applications. It is not surprising that *Geobacillus* sp. 12AMOR1 was isolated from a 90 °C hot deep-sea sediment sample and was expected to produce enzymes with great tolerance against high temperature. It has been reported that some lipases derived from the marine microorganism possessed great thermostable properties, whereas some of them were cold adaptive (Table 2).

#### 2.2.2. Effect of pH on Lipase Activity of GMGL

**Figure 4:**
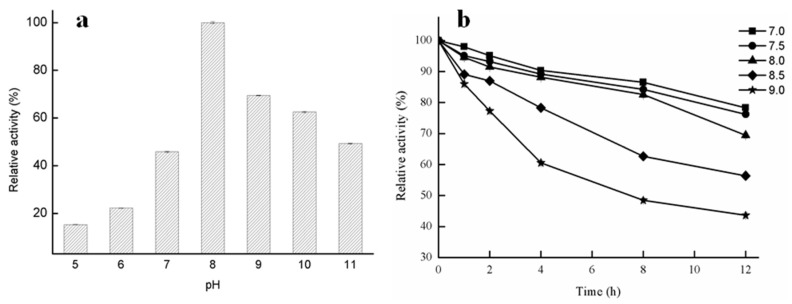
Effect of pH on activity of GMGL. The GMGL activities under various pH were determined using glycerin monostearate as substrate. The activity obtained at pH 8.0 was taken as 100%.

The effects of pH on the enzymatic activity of GMGL were shown in Figure 4. The GMGL was found to have a relatively high activity at alkaline pH condition, and display the highest activity at pH 8. It retained about 43% to 64% activity at pH ranging from 9 to 11. In contrast, its activity sharply dropped to below 22% at pH 5 and 6. For long-term (12 h) incubation of the enzyme in pH 7.0–8.0 buffer, GMGL still had more than 70% of the enzyme activity. At pH 8.5 and 9.0, the enzyme activity decreased much faster within the first four hours, leaving only 78% and 60% of residual enzyme activity, respectively (Figure 4b).

#### 2.2.3. Effects of Metal Ions and Solvents on Lipase Activity of GMGL

**Figure 5:**
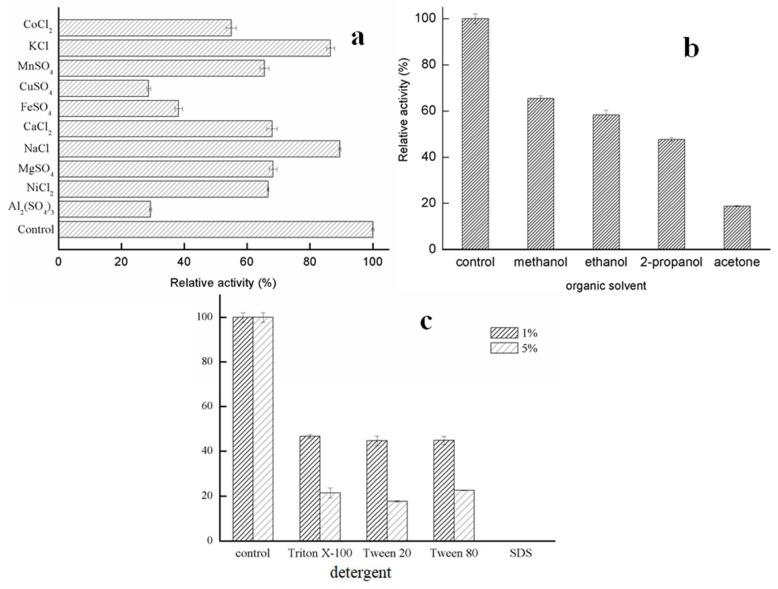
Activity of GMGL the influence of ions and solvents. (**a**) Effects of different metal ions on GMGL activity. (**b**) Effects of different organic solvents on the enzyme activity. (**c**) Effects of different concentration of different detergents. The GMGL activities were determined in the presence of various compounds using pNP-C8 as substrate. Sample without adding any compounds was used as control and its activity was taken as 100%.

Metal ions were found to present inhibitory effects on GMGL activity to different extents. GMGL was stable in the presence of K^+^ and Na^+^, with more than 80% of the original activity, while Mn^2+^, Ca^2+^, Mg^2+^ and Ni^2+^ showed weak inhibitory effects on the activity of GMGL. In contrast, Cu^2+^, Fe^2+^ and Al^3+^ exhibited stronger inhibitory effects, with the relative enzyme activity dropping to 28.69%, 38.22% and 29.31%, respectively (Figure 5a). One of the explanations might be that these metal ions might cause structural changes of the enzyme, which are unfavorable for substrate processing. 

Organic solvents are often used as the component in the catalytic reaction for practical applications [19]. Thus the tolerances of GMGL against organic solvents were investigated. The activity of GMGL was assessed in the presence of 10% concentration of these organic solvents. As shown in Figure 5, organic solvents can decrease activity of GMGL. The enzyme maintained about 60% relative activity of control when treated with methanol and ethanol, while GMGL sharply decreased its activity with the residual activity of 18.83% in the presence of acetone (Figure 5).

Detergents can change the proportion of lipid in the interface of oil and water, affecting the access of substrate into the active site of enzyme [20]. The activity of GMGL was determined in the presence of 1% and 5% concentrations of several detergents, respectively. All detergents tested in this study seriously decreased the activity of GMGL, and the inhibitory profiles of Triton X-100, Tween 20 and Tween 80 were similar. The GMGL retained about 45% and 20% relative activity in the presence of 1% and 5% concentration of these detergents, respectively. Complete inhibition of GMGL by sodium dodecyl sulfate (SDS) was observed (Figure 5c).

#### 2.2.4. Substrate Specificity of GMGL

**Figure 6:**
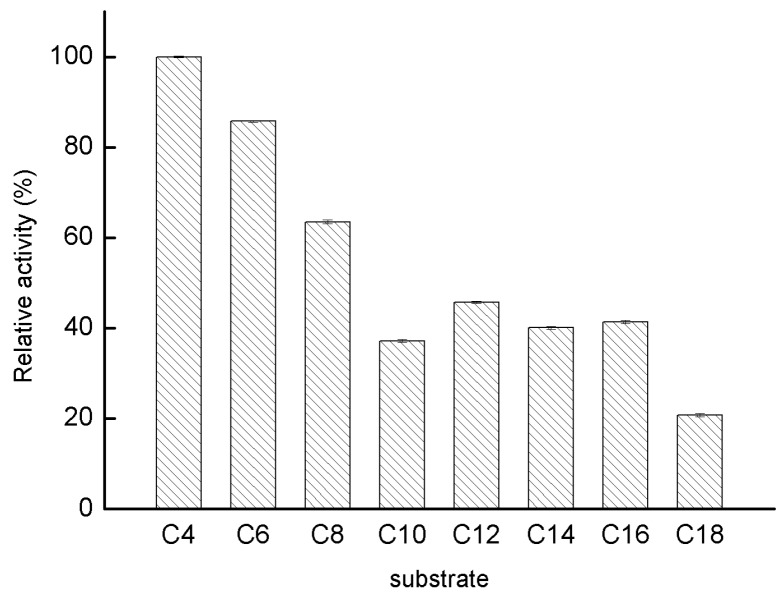
Substrate specificity of GMGL. The activities of GMGL toward pNP-esters with various acyl-chain lengths were determined. The activity obtained using pNP-C4 was taken as 100%.

Initially, pNP-esters with various acyl-chain lengths ranging from C4 to C18 were chosen to probe the substrate specificity of GMGL. As shown in Figure 6, GMGL showed a preference toward pNP-esters with shorter acyl-chains. GMGL showed the highest activity toward pNP-C4. The relative activities toward the pNP-esters with medium and long acyl-chain lengths (C10 to C18) were below 45%.

**Figure 7:**
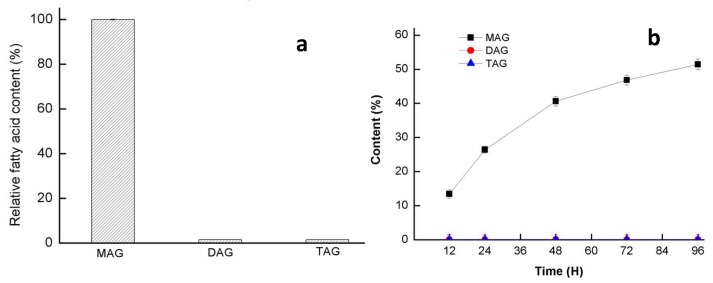
Hydrolysis (**a**) and synthesis (**b**) activity of GMGL. (**a**) The hydrolysis activity of GMGL toward glycerin monostearate (MAG), 2,2-Dilauric acid glyceride (DAG) and triolein a (TAG) were assessed. The activity on MAG was taken as 100%. (**b**) The esterification activity of GMGL was measured using glycerol and linolenic acid as substrate. Reaction conditions were as follows: glycerol/linolenic acid molar ratio, 4:1; lyophilization GMGL, 20 mg; initial water content, 2% *w*/*w*, with respect to total reaction mixture; agitation speed, 500 rpm; reaction time, 96 h.

Then natural oil substrate (glycerin monostearate, triolein and 2,2-Dilauric acid glyceride) were used to confirm its glyceride selectivity (Figure 7). GMGL showed restrict activity toward MAG substrate, while it cannot hydrolyze the DAG and TAG substrate event for a longer reaction time. To further confirm whether GMGL belong to the group of monoacylglycerol, its ability to produce MAG were assessed. Esterification using glycerol and linolenic acid as substrate was conducted. Only MAG products were found in the reaction after 96 h reaction. The hydrolysis and synthesis activity test both confirmed that GMGL was a truly MGL.

### 2.3. Structure Analysis of GMGL

#### 2.3.1. Structure Model of GMGL

**Figure 8:**
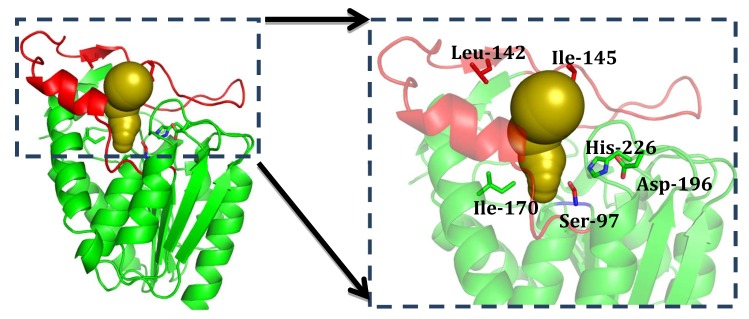
The structure model of GMGL and substrate binding pathway illustration. Cartoon view of GMGL catalytic pocket with substrates tunnel (Golden) and cap region (Red) highlighted. Residues were shown in stick model. Leu142 and Ile145 located in the cap region and Ile170 sited at the catalytic pocket.

To get more structural detail, homology model of GMGL was constructed. The crystal structure of bMGL has been previously resolved and was used as the template in this study due to its relatively high sequence identity with GMGL. According to the homology model, GMGL displays the canonical architecture of an α/β-hydrolase fold consisting of eight α-helices, nine β-strands and loops connecting these α-helices and β-strands. According to the structure comparison and sequence alignment with other MGLs, the catalytic triad of GMGL was found to be made up of Ser97, Asp196 and His226 (Figure 8). The active site was located in the bottom of a long narrow substrate-binding tunnel. By contrast with the common alpha helix lid domain, GMGL possesses a cap domain consisting of one α-helix and two small antiparallel β-sheets, which can adjust its conformation from the close to open state to control the access of the substrate into the active site [27].

#### 2.3.2. Molecular Basis for the Acyl Chain Length Selectivity

**Figure 9:**
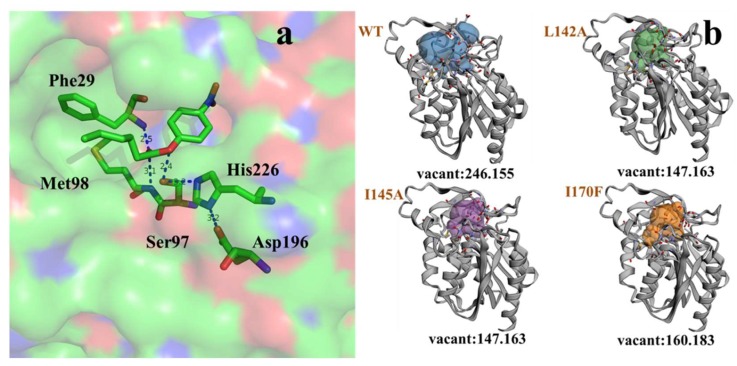
Docking of pNP-C6 with GMGL (**a**) and catalytic cavity changes between wild type (WT) and mutations (**b**).

Study of the acyl-chain length profile of GMGL showed that GMGL prefers to hydrolyze short acyl chain length pNP-esters. To probe the molecular basis of substrate specificity of GMGL, a mutagenesis study on the substrate binding tunnel was performed. Leu142, Ile145 located at cap domain and Ile170 sited at the catalytic pocket were selected for this study. Substitution of Leu 142 and Ile145 with Ala might change the geometry of the entrance of the substrate-binding pocket allowing access of substrate with longer acyl-chain mutation of Ile170 to Phe might affect the hydrophobic interaction of this site with the substrate, resulting in enhanced binding. As summarized in Table 3, the k_cat_/K_m_ value toward pNP-C6 in mutants Leu142Ala, Ile145Ala and Ile170Phe increased to 2.3-, 1.4- and 2.2-fold as compared to that of GMGL wild type (WT), respectively. Interestingly, all GMGL mutants had a lower K_m_ value toward pNP-C6 than that of the GMGL WT, implying that GMGL increase its affinity to this substrate after mutation. Comparison of catalytic cavity GMGL WT with mutants showed that the GMGL mutants have a smaller catalytic chamber volume than that of GMGL WT (Figure 9), which might enhance the hydrophobic interaction between the catalytic cavities with the substrate. Previous studies reported that modification of the volume, shape, and hydrophobicity of this cavity may affect the acyl chain-binding energy resulting in changes of the substrate preference [28,29].

## 3. Materials and Methods

### 3.1. Materials

*Escherichia coli* (*E. coli*) Top10 and plasmid pET-30a (+) (Invitrogen, Shanghai, China) were used as cloning host and vector, respectively. *E. coli* BL21(DE3) strain (Invitrogen, Shanghai, China) was used for protein expression. A SanPrep Column Plasmid Mini-Preps Kit purchased from Sangon Biotech (Shanghai, China) was used to extract plasmids. Modified Bradford Protein Assay Kit (Sangon, Shanghai, China) was used to measure protein concentration. The molecular marker (Code No. 3595Q) was obtained from Takara (Dalian, China). The glycerin monostearate rich oil (≥78%) was synthesized in our laboratory. Glycerol, linolenic acid (~70%), 4-Nitrophenol solution, pNP-C4 to C18 were obtained from Sigma-Aldrich (Shanghai, China). All other chemicals and reagents used in the study were of analytical grade.

### 3.2. Expression and Purification of GMGL

The putative MGL gene sequence (GenBank Accession number: AKM18206.1) was discovered from the genomic data of thermophilic *Geobacillus* sp. 12AMOR1 in an Arctic deep-sea hydrothermal vent site. The gene was obtained by direct synthesis from Sangon Biotech (Shanghai, China). Subsequently, GMGL was cloned into the pET-30a (+) expression vector in the location between EcoRI and XhoI restriction enzyme sites and fused with a C-terminal His-tag. Mutagenesis of GMGL gene was performed following a published protocol [30]. The plasmid pET-30a (+)-GMGL was used as the template and primers used for gene mutagenesis were listed in Table 4. All the plasmids contained the GMGL gene was transformed into bacterial host for expression.

*E. coli* BL21(DE3) cells harboring expression plasmids were grown in LB broth (1% Tryptone, 0.5% NaCl, 0.5% Yeast extract) at 37 °C from an overnight seed culture until the optical density (OD600) reaches 0.6-0.8. The culture was induced by 0.2 mM isopropyl β-d-1-thiogalactopyranoside (IPTG) and further incubated for 20 h at 20 °C. The cells were subsequently harvested and lysed by ultrasonication in phosphate buffered saline (PBS, 137 mM NaCl, 2.7 mM KCl, 10 mM Na_2_HPO_4_, 2 mM KH_2_PO_4_, pH 7.4). The parameters for ultrasonication were set as two sec on, four sec off, with a total working time of 15 min under 80 watts. The lysate was then centrifuged at 12,000 rpm for 30 min and the soluble fraction was loaded into a HisPrep^TM^ FF 16/10 column (GE Healthcare, Uppsala, Sweden). The target protein was eluted with 500 mM NaCl, 500 mM imidazole, 20 mM Tris-HCl pH 8.0 and the purified protein was stored in buffer A (20 mM Tris-HCl pH 8.0). The purity of the protein was analyzed by 12% sodium dodecyl sulfate polyacrylamide gel electrophoresis (SDS-PAGE, prepared in-house) and the protein concentration was determined by BCA Protein Assay Kit from Sangon Biotech (Shanghai, China) according to the manual from the manufacturer.

### 3.3. Biochemical Characterization of GMGL

#### 3.3.1. Activity Determination

GMGL activity was assessed with pNP-ester and natural oil as substrates. For pNP-ester substrate, the reaction mixture consisted of 80 μL buffer (20 mM Tris-HCl, pH 8.0), 10 μL enzyme in appropriate concentration, 10 μL pNP-ester in 10 mM (dissolved in ethanol). The reaction mixture was incubated at 30 °C for 10 min, and terminated by adding 100 μL 1% SDS. The absorbance of the reaction mixture was measured at 405 nm. One unit of enzyme activity is defined as the amount of enzyme required for releasing one μmol of p-nitrophenol per minute.

For natural oil substrates, the hydrolytic reactions were carried out in a 50 mL conical flask with stirring at 500 rpm. The hydrolysis of glycerin monostearate emulation (one gram of glycerin monostearate mixed with three grams of 4% polyvinyl alcohol) was measured on the condition of buffer-20 mM Tris-HCl, pH 8.0- (5 g/g, with respect to substrate), purified lipases (1 mg/g, with respect to substrate) at the optimum temperature (60 °C) for 10 min. The hydrolysis reaction was stopped by adding 15 mL 95% ethyl alcohol. The amount of released fatty acids was calculated through titration with NaOH (0.05 mol/L). One unit is defined as the amount of enzyme which released one micromole of fatty acid in one min.

#### 3.3.2. Effects of Temperature on the Lipase Activity of GMGL

The optimal temperature of the lipase activity was measured at pH 7.0 using glycerin monostearate as substrate. The temperature range was from 30 to 80 °C. The thermostability of the lipase GMGL was determined by preincubating the purified solution at different temperatures, and the samples were taken every 30 min for measurement of residual activity with the method of lipase activity assay until 90 min. The temperature was set as 50, 55, 60, 65 and 70 °C.

#### 3.3.3. Effect of pH on Lipase Activity of GMGL

Buffer pH range was from 5.0 to 11.0 (pH 5.0, 0.1 M sodium citrate, 0.1 M citric acid; pH 6.0–7.0, 0.1 M phosphate buffer; pH 8.0, 0.05 M Tris-HCl; pH 9.0–10.0, 0.05 M Glycine-NaOH; pH 11.0, 0.025 M Na_2_CO_3_-NaOH). Optimal pH for the purified GMGL lipase activity was measured at 60 °C with the method of lipase activity assay using glycerin monostearate as substrate.

#### 3.3.4. Substrate Specificity of GMGL Lipase

Substrate specificity for GMGL was researched using 10 mM pNP ester that was dissolved in ethanol with different chains length: pNP-C4, pNP-C6, pNP-C8, pNP-C10, pNP-C12, pNP-C14, pNP-C16 and pNP-C18. The activities toward different substrate were measured according to standard method described above.

#### 3.3.5. Effect of Metal Ions, Detergents and Organic Solvents on the Lipase Activity of GMGL

Various metal ions (ZnSO_4_, CuSO_4_, MgSO_4_, CaCl_2_, NiCl_2_, MnSO_4_, MnCl_2_, K2SO_4_, MgCl_2_ and NaCl) at final concentrations of 1 mM were added to the enzyme in pH 8.0, and then the lipase activity was assayed after incubating at 4 °C for 2 h. The effect of detergents (Triton X-100, Tween 20, Tween 80 and SDS) and organic solvents (methanol, ethanol, 2-propanol and acetone) on the lipase activity was determined by incubating the enzyme with 10% (*v*/*v*) of the different organic solvents and 1%, 5% detergents for 2 h at 4 °C and pH 8.0. The lipase activity was measured at the beginning and end of the incubation period using pNP-C8 as substrate. The enzyme activity in the absence of detergent and organic solvent was defined as the 100%.

#### 3.3.6. Determination of Enzyme Kinetic Parameters

Different concentration of pNP-ester substrate (pNP-C4, pNP-C6 and pNP-C8) ranging from 0.05 to 10 mM was prepared. The reaction consisted of 10 µL pNP-ester liquid, 10 µL liquid containing GMGL or its mutants and 180 µL reaction buffer (20 mM Tris-HCl, pH 8.0). The heat-inactive enzyme was used as control. The kinetic reaction process was monitored by Tecan Infinite 200 Pro (Tecan, Switzerland) with the followed program: kinetic duration, 5 s; interval time, 10 s; total time, 10 min; measurement wavelength, 405 nm; temperature, 30 °C. The kinetic constants (k_cat_, K_m_, and k_cat_/K_m_) were calculated by fitting the initial rate data into the Michaelis–Menten equation in GraphPad Prism 6. 

### 3.4. Hydrolysis Activity of Triacylglycerol (TAG) and 2,2-Dilauric Acid Glyceride (DAG)

The hydrolytic reactions were carried out in a 10mL conical flask with stirring at 500 rpm. The hydrolysis of TAG and DAG was measured on the condition of water (25% content with respect to substrate), purified lipase (3600 U/g, with respect to substrate) at the optimal temperature (55 °C) for 24 h, and aliquots (200 µL) of the reaction mixture were periodically withdrawn were diluted in 1 mL of n-hexane/2-propanol/methanoic acid (21:1:0.003 *v*/*v*/*v*) for high-performance liquid chromatography (HPLC) analysis. Sodium sulfate without water were added and then centrifuged at 12,000 rpm for 2 min to remove the water in the upper layer.

The contents of acyl glycerol and FFAs in the hydrolytic products were analyzed using a 2695 HPLC (Waters, Milford, MA, USA) with a 2414 parallax refractive index detector (Waters) on a Phenomenex Luna silica column (Phenomenex Corporation, Torrance, CA, USA; 250 × 4.6 mm^2^ i.d., 5 µm particle size). The mobile phase was a mixture of n-hexane/2-propanol/formic acid (21:1:0.003 *v*/*v*/*v*) with a flow rate of 1.0 mL/min. Peaks in HPLC were identified by comparison of the retention times with reference standards. Peak areas percentages were calculated using a 2695 integration software (Waters). All the experiments were replicated triple and the results presented were the mean values for the replicated data.

### 3.5. Esterification Activity of GMGL

To test the esterification ability of GMGL, reactions using glycerol and linolenic acid as substrate were conducted. The reaction mixture in a flask contained 0.64 g linolenic acid, 1.36 g glycerol, 20 mg lyophilization enzyme of GMGL WT and buffer (20 mM Tris-HCl, 20 mM NaCl, pH 8.0, (2% *w*/*w*, with respect to total reaction mixture). The flask was incubated in a glycerol bath of 40 °C having a magnet stick with a speed of 500 rpm. The samples from the reaction were withdrawn at periodic intervals and analyzed with HPLC. Peak-area percentages were obtained by the Waters Breeze 2 software using an area normalization method. The MAG content was calculated as the percentage of MAG peak area vs. the sum of peak area of MAG and fatty acid.

### 3.6. Homology Modeling and Docking Analysis

The three-dimensional protein structure of GMGL was constructed with the SWISS-MODEL workspace. bMGL protein sequence (PDB ID:4KE6) was selected as the automated homology modeling template because of the highest protein sequence identity between bMGL and GMGL [31,32]. Following the operational instructions of SWISS-MODEL, GMGL structure models were generated. Docking analysis of GMGL with pNP-ester was performed by the AutoDock 4.0 program, using the implemented empirical free energy function and the Lamarckian genetic algorithm (LGA) [33,34]. The grid maps were calculated using AutoGrid. In all dockings, a grid map with 58 × 42 × 40 points and a grid-point spacing of 0.375 Å was applied. Because the location of the ligand in the complex was known, the maps were centered on the ligand’s binding site on OH^−^ of Ser97. For all docking parameters, standard values were used as described before. The ligand pose with best position and orientation was chosen as the model for further analysis. 

## 4. Conclusions

A novel thermostable monoacylglycerol lipase from marine *Geobacillus* sp. 12AMOR1 has been characterized in this work. The recombinant GMGL has been overexpressed in *E. coli* and isolated by affinity chromatography with a total activity of 163,812 U from a one liter fermentation broth. GMGL is a thermostable enzyme with high activity at 60 °C, and shows specific activity toward MAG but not DAG or TAG. After mutagenesis in substrate binding pocket, the k_cat_/K_m_ values toward pNP-C6 substrate in mutants Leu142Ala, Ile145Ala and Ile170Phe increased 2.3-, 1.4- and 2.2-fold compared to that of GMGL WT, respectively. Our work characterized a novel monoacylglyerol lipase from a marine source, which shed some light on understanding the substrate selectivity of monoacylglycerol lipases.

## Figures and Tables

**Table 1:** Summary of purification of GMGL.

Fraction	Total Activity (U)	Total Protein (mg)	Specific Activity (U/mg)	Purification Fold	Yield (%)
Lysate	178,080	1484.8	120	1	100
Affinity chromatography	163,812	264	620.5	4.85	17.7

**Table 2:** Summary of biochemical properties of lipases from marine microorganisms.

Entry	Species	Enzyme	Substrate	pH	Temperature (°C)	Thermostability (Half-Life)	Reference
1	*Geobacillus* sp. 12AMOR1	GMGL	Glycerin monostearate	8.0	60	60 min at 70 °C	This work
2	*Streptomyces* sp. strain W007	MAS1	Camellia oil	7.0	40	90 min at 70 °C	[15]
3	*Janibacter* sp. strain HTCC2649	MAJ1	DAG and MAG in Camellia oil	7.0	30	less than 30 min at 40 °C	[21]
4	*Haliclona simulans*	Lpc53E1	pNP-ester	7.0	40	60 min at 90 °C	[22]
5	*Yarrowia lipolytica* yeast	LIPY	pNP-ester	8.5	35	more than 120 min at 70 °C	[23]
6	*Psychrobacter* sp.	MBP-lipase	pNP-ester	9	20	10 min at 40 °C	[24]
7	*Pseudomonas* sp. 7323	rLipA.	pNP-ester	9	30	4.5 h at 30 °C	[25]
8	Uncultured bacteria of marine sediment	h1Lip1	pNP-ester	7.25	35	less than 5 min at 40 °C	[26]

**Table 3:** Enzyme kinetics of GMGL WT and mutations.

Ester	WT			Leu142Ala			Ile145Ala			Ile170Phe		
Kinetic Parameter	K_m_ (mM)	k_cat_ (S^−1^)	k_cat_/K_m_ (mM^−1^·S^−1^)	K_m_ (mM)	k_cat_ (S^−1^)	k_cat_/K_m_ (mM^−1^·S^−1^)	K_m_ (mM)	k_cat_ (S^−1^)	k_cat_/K_m_ (mM^−1^·S^−1^)	K_m_ (mM)	k_cat_ (S^−1^)	k_cat_/K_m_ (mM^−1^·S^−1^)
C4	6.80	9.74	1.43	14.78	15.89	1.08	9.56	3.25	0.34	2.37	3.31	1.21
C6	4.06	4.31	1.06	1.66	4.08	2.46	1.34	2.02	1.51	1.60	3.67	2.29
C8	2.83	2.58	0.91	4.65	1.29	0.28	8.32	0.95	0.11	4.63	2.08	0.45

**Table 4:** Primers using for the mutation construction.

Mutations	Forward/Reverse Primers
Leu142A	F: GATCTGCCGCGTTTCGCCGATGCAATCGGTTC R: GAACCGATTGCATCGGCGAAACGCGGCAGATC
Ile145Ala	F: CCGCGTTTCCTGGATGCAGCAGGTTCCGATATCAAAAAAC R: GTTTTTTGATATCGGAACCTGCTGCATCCAGGAAACGCGG
Ile170Phe	F: CAAGCATCCGTCAGTTCGTTCAGCTGATG R: CATCAGCTGAACGAACTGACGGATGCTTG

## Abbreviations

GMGL	*Geobacillus* sp. monoacylglycerol Lipase
MGL	Monoacylglycerol Lipase
MAG	Monoacylglycerol
DAG	Diacylglycerol
TAG	Triacylglycerol
WT	Wild type
IPTG	Isopropyl β-d-Thiogalactoside
pNP-C4	4-Nitrophenyl butyrate
pNP-C6	4-Nitrophenyl caproate
pNP-C8	4-Nitrophenyl octanoate
pNP-C10	4-Nitrophenyl decanoate
pNP-C12	4-Nitrophenyl laurate
pNP-C14	4-Nitrophenyl myristate
pNP-C16	4-Nitrophenyl palmitate
pNP-C18	4-Nitrophenyl stearate

## References

[1] Rudkowska I., Roynette C.E., Demonty I., Vanstone C.A., Jew S., Jones P.J. (2005). Diacylglycerol: Efficacy and mechanism of action of an anti-obesity agent. Obes. Res..

[2] Khaddaj-Mallat R., Morin C., Rousseau E. (2016). Novel n-3 PUFA monoacylglycerides of pharmacological and medicinal interest: Anti-inflammatory and anti-proliferative effects. Eur. J. Pharmacol..

[3] von der Haar D., Stabler A., Wichmann R., Schweiggert-Weisz U. (2015). Enzyme-assisted process for DAG synthesis in edible oils. Food Chem..

[4] Cotes K., Dhouib R., Douchet I., Chahinian H., de Caro A., Carriere F., Canaan S. (2007). Characterization of an exported monoglyceride lipase from *Mycobacterium tuberculosis* possibly involved in the metabolism of host cell membrane lipids. Biochem. J..

[5] McPherson J.C., Askins R.E., Pope J.L. (1962). The specificity of an intestinal lipase for monoglycerides. Exp. Biol. Med..

[6] Aschauer P., Zimmermann R., Breinbauer R., Pavkov-Keller T., Oberer M. (2018). The crystal structure of monoacylglycerol lipase from *M. tuberculosis* reveals the basis for specific inhibition. Sci. Rep..

[7] Aschauer P., Rengachari S., Lichtenegger J., Schittmayer M., Das K.M., Mayer N., Breinbauer R., Birner-Gruenberger R., Gruber C.C., Zimmermann R. (2016). Crystal structure of the *Saccharomyces cerevisiae* monoglyceride lipase Yju3p. Biochim. Biophys. Acta..

[8] Bertrand T., Auge F., Houtmann J., Rak A., Vallee F., Mikol V., Berne P.F., Michot N., Cheuret D., Hoornaert C. (2010). Structural basis for human monoglyceride lipase inhibition. J. Mol. Biol..

[9] Rengachari S., Bezerra G.A., Riegler-Berket L., Gruber C.C., Sturm C., Taschler U., Boeszoermenyi A., Dreveny I., Zimmermann R., Gruber K. (2012). The structure of monoacylglycerol lipase from *Bacillus* sp. H257 reveals unexpected conservation of the cap architecture between bacterial and human enzymes. Biochim. Biophys. Acta..

[10] Derewenda U., Brzozowski A.M., Lawson D.M., Derewenda Z.S. (1992). Catalysis at the interface the anatomy of a conformational change in a triglyceride lipase. Biochemistry.

[11] Brzozowski A.M., Derewenda U., Derewenda Z.S., Dodson G.G., Lawson D.M., Turkenburg J.P., Bjorkling F., Huge-Jensen B., Patkar S.A., Thim L. (1991). A model for interfacial activation in lipases from the structure of a fungal lipase-inhibitor complex. Nature.

[12] Gurung N., Ray S., Bose S., Rai V. (2013). A broader view: Microbial enzymes and their relevance in industries, medicine, and beyond. BioMed. Res. Int..

[13] Siddiqui K.S., Poljak A., Guilhaus M., De Francisci D., Curmi P.M., Feller G., D’Amico S., Gerday C., Uversky V.N., Cavicchioli R. (2006). Role of lysine versus arginine in enzyme cold-adaptation: Modifying lysine to homo-arginine stabilizes the cold-adapted α-amylase from *Pseudoalteramonas haloplanktis*. Proteins.

[14] Trincone A. (2011). Marine biocatalysts: Enzymatic features and applications. Marine Drugs.

[15] Yuan D., Lan D., Xin R., Yang B., Wang Y. (2016). Screening and characterization of a thermostable lipase from marine, *Streptomyces*, sp. strain W007. Biotechnol. Appl. Biochem..

[16] Wang X., Li D., Qu M., Durrani R., Yang B., Wang Y. (2017). Immobilized MAS1 lipase showed high esterification activity in the production of triacylglycerols with n-3 polyunsaturated fatty acids. Food Chem..

[17] Wang X., Li D., Wang W., Yang B., Wang Y. (2016). A highly efficient immobilized MAS1 lipase for the glycerolysis reaction of n-3 PUFA-rich ethyl esters. J. Mol. Catal. B.

[18] Wissuwa J., Stokke R., Fedoy A.E., Lian K., Smalas A.O., Steen I.H. (2016). Isolation and complete genome sequence of the thermophilic *Geobacillus* sp. 12AMOR1 from an Arctic deep-sea hydrothermal vent site. Stand Genom. Sci..

[19] Li D., Wang W., Durrani R., Li X., Yang B., Wang Y. (2016). Simplified Enzymatic Upgrading of High-Acid Rice Bran Oil Using Ethanol as a Novel Acyl Acceptor. J. Agric. Food Chem..

[20] Mogensen J.E., Sehgal P., Otzen D.E. (2005). Activation, inhibition, and destabilization of *Thermomyces lanuginosus* lipase by detergents. Biochemistry.

[21] Yuan D., Lan D., Xin R., Yang B., Wang Y. (2014). Biochemical properties of a new cold-active mono- and diacylglycerol lipase from marine member *Janibacter* sp. strain HTCC2649. Int. J. Mol. Sci..

[22] Selvin J., Kennedy J., Lejon D.P., Kiran G.S., Dobson A.D. (2012). Isolation identification and biochemical characterization of a novel halo-tolerant lipase from the metagenome of the marine sponge Haliclona simulans. Microb. Cell Fact..

[23] Sheng J., Wang F., Wang H., Sun M. (2012). Cloning, characterization and expression of a novel lipase gene from marine psychrotrophic *Yarrowia lipolytica*. Ann. Microbiol..

[24] Parra L.P., Reyes F., Acevedo J.P., Salazar O., Andrews B.A., Asenjo J.A. (2008). Cloning and fusion expression of a cold-active lipase from marine Antarctic origin. Enzym. Microb. Technol..

[25] Zhang J.-W., Zeng R.-Y. (2008). Molecular cloning and expression of a cold-adapted lipase gene from an Antarctic deep sea psychrotrophic bacterium *Pseudomonas* sp. 7323. Mar. Biotechnol..

[26] Hårdeman F., Sjöling S. (2007). Metagenomic approach for the isolation of a novel low-temperature-active lipase from uncultured bacteria of marine sediment. FEMS Microbiol. Ecol..

[27] Khan F.I., Lan D., Durrani R., Huan W., Zhao Z., Wang Y. (2017). The Lid Domain in Lipases: Structural and Functional Determinant of Enzymatic Properties. Front. Bioeng. Biotechnol..

[28] Wang Y., Ryu B.H., Yoo W., Lee C.W., Kim K.K., Lee J.H., Kim T.D. (2018). Identification, characterization, immobilization, and mutational analysis of a novel acetylesterase with industrial potential (LaAcE) from *Lactobacillus acidophilus*. Biochim. Biophys. Acta. Gen. Subj..

[29] Jing F., Zhao L., Yandeau-Nelson M.D., Nikolau B.J. (2018). Two distinct domains contribute to the substrate acyl chain length selectivity of plant acyl-ACP thioesterase. Nat. Commun..

[30] Storici F., Lewis L.K., Resnick M.A. (2001). In vivo site-directed mutagenesis using oligonucleotides. Nat. Biotechnol..

[31] Kitaura S., Suzuki K., Imamura S (2001). Monoacylglycerol lipase from moderately thermophilic *Bacillus* sp. strain H-257: Molecular cloning, sequencing, and expression in *Escherichia coli* of the gene. J. Biochem..

[32] Imamura S., Kitaura S. (2000). Purification and characterization of a monoacylglycerol lipase from the moderately thermophilic *bacillus* sp. H-257. J. Biochem..

[33] Morris G.M., Goodsell D.S., Halliday R.S., Huey R., Hart W.E., Belew R.K., Olson A.J. (1998). Automated docking using a Lamarckian genetic algorithm and an empirical binding free energy function. J. Comput. Chem..

[34] Bordoli L., Kiefer F., Arnold K., Benkert P., Battey J., Schwede T. (2009). Protein structure homology modeling using SWISS-MODEL workspace. Nat. Protoc..

